# Network modeling of sporadic colorectal cancer reveals the importance of off-target effects of Cyclooxygenase inhibitors

**DOI:** 10.1038/s41540-025-00622-x

**Published:** 2026-02-27

**Authors:** Anthony R. Gebhart, Manon M. M. Berns, Jan Snoeys, Jeroen Elassaiss-Schaap, Elizabeth C. M. de Lange

**Affiliations:** 1 Research Division of Systems Pharmacology and Pharmacy, LACDR, Leiden University, Leiden, The Netherlands; 2 PD-value B.V., Utrecht, The Netherlands; 3 Translational Pharmacokinetics Pharmacodynamics and Investigative Toxicology, Johnson & Johnson, Beerse, Belgium

**Keywords:** Cancer, Cell biology, Drug discovery, Systems biology

## Abstract

Cyclooxygenase (COX) inhibitors (COXIBs) have shown preventive and therapeutic potential for colorectal cancer (CRC). In addition to inhibiting COX, approved COXIBs also target COX-independent pathways, including NF-κB and AKT. We evaluated how inhibition of various COXIB targets affects cell proliferation and apoptosis using a Boolean model incorporating the effect of several genetic mutations linked to CRC. The mutations activate two positive feedback loops and cause increased cell proliferation and survival. When simulating loss of function of APC, proliferation and apoptosis can be restored to healthy rates by inhibiting COX2. In simulations reflecting other genetic mutations, none of the investigated targets restore apoptosis and proliferation is reduced by inhibiting AKT or NF-κB, but not by any of the other COX targets tested. These results show that the off-target effects of COXIBs are crucial for their efficacy in the treatment of sporadic CRC.

## Introduction

Colorectal Cancer (CRC) is one of the most prevalent cancers and a significant cause of mortality worldwide^1^. Despite recent advances in the development of targeted therapies, the overall five-year survival of patients with metastatic cancer is less than 20%^2^. Hence, there is a need for the development of new treatment approaches for CRC.

Inflammation plays a prominent role in CRC carcinogenesis^3^. Therefore, nonsteroidal anti-inflammatory drugs (NSAIDs), and specifically cyclooxygenase (COX) inhibitors (COXIBs), have gained significant attention in the prevention and treatment of CRC^4^. The COXIBs sulindac and celecoxib have been shown to reduce the incidence of polyps in mice^5–10^. Moreover, clinical studies have shown the efficacy of celecoxib in preventing the formation of colorectal adenomas in subjects who underwent surgical removal of adenomas^11,12^. Also, in patients with familial adenomatous polyposis (FAP), a germline mutation initiating CRC development^13^, celecoxib reduced adenoma development^14^. Despite these positive results, the use of COXIBs to prevent CRC is controversial because of cardiovascular safety concerns and increased risk of serious bleeding events^15^. In later stages of CRC development, the effectiveness of celecoxib is uncertain. Celecoxib in combination with chemotherapy has been reported to increase overall survival of patients with metastatic CRC^16^, whereas celecoxib as adjuvant therapy did not increase the disease-free and overall survival of patients with stage III colon cancer^17^. Moreover, multiple clinical studies examining COXIBs as neoadjuvant therapy in the treatment of CRC have been stopped because of severe cardiovascular adverse effects^4^. Improving the efficacy and safety of COXIBs in CRC treatment requires a deeper understanding of the mechanisms of action of COXIBs in the context of genetic mutations commonly encountered in CRC.

COXIBs function through inhibition of COX, an enzyme that converts arachidonic acids into prostaglandins such as prostaglandin E_2_ (PGE2). There are three COX isoforms, COX1, 2, and 3. COX1 and COX3 are housekeeping genes expressed in healthy tissue^18^. COX2 is generally expressed at low levels, but its expression increases during inflammation^18^. COX2 expression is found to be elevated in CRC tumors^19,20^. In adenomatous-polyposis-coli (APC) knockout mice, a mouse model of FAP, inactivating the COX2 gene resulted in a reduction in the size and number of intestinal polyps^19^. This shows that COX2 plays an important tumor-promoting role in CRC.

Besides inhibiting COX, COXIBs, including celecoxib and sulindac, have various off-target effects^21,22^. This is illustrated by the ability of celecoxib to induce cell death in a cancer cell line lacking COX2 catalytic activity^23^. The sulindac metabolite sulindac sulfide and celecoxib have been shown to elevate the intracellular cGMP levels via inhibition of phosphodiesterases (PDEs), and more specifically, PDE5^24,25^. The elevated intracellular cGMP levels activated protein kinase G (PKG) and reduced transcriptional activity of β-catenin, which is typically highly active in CRC^24^. β-catenin activity is also inhibited by sulindac via an inhibition of the interaction between Dishevelled (DVL) and the Wnt-receptor Frizzled (FZD)^26^. Another important COX-independent target of celecoxib is the AKT pathway, which is commonly hyperactive in cancers and plays an important role in regulating pro-proliferative signaling^27,28^. Celecoxib inhibits the phosphorylation of AKT, and thereby its activation, through inhibiting PDK1^23,29^. The COXIBs sulindac and celecoxib are also proposed to inhibit the transcriptional activity of NF-κB^30,31^. It is uncertain whether this occurs via AKT or is mediated by a direct interaction between the COXIB and NF-κB. Lastly, COXIBs have been suggested to interact with BCL-2 family proteins, which regulate apoptosis^32^. The importance of the various COXIBs targets to the overall effectiveness of COXIBs in CRC treatment is uncertain and may be strongly dependent on the specific mutations present in a given tumor. In sporadic CRC, which occurs in patients without a known genetic predisposition or family history, frequently observed genetic alterations include loss of function (LOF) mutations in APC and p53, gain of function (GOF) mutations in RAS, and allelic loss at chromosome 18q^33,34^.

In this work, we developed a Boolean model to investigate the interplay between genetic mutations frequently associated with sporadic CRCs and the inhibition of different proteins through the action of COXIBs. Within the sporadic CRC (spoCRC) network, we identified two positive feedback loops, which, in the presence of inflammation, lead to aberrant proliferative and apoptotic signaling. We found that mutations directly or indirectly inducing the activation of one of the proteins within these positive feedback loops cause a shift to a pro-proliferative phenotype. Analyzing the effect of inhibition of the proteins targeted by COXIBs, we observed that COX2 inhibition restores proliferation and apoptosis to healthy rates in those cases in which APC function is lost. Interestingly, in the simulations incorporating the effect of any of the other mutations, inhibition of AKT is most efficient in reducing cancer cell proliferation.

## Results

### The spoCRC network

**Fig. 1:**
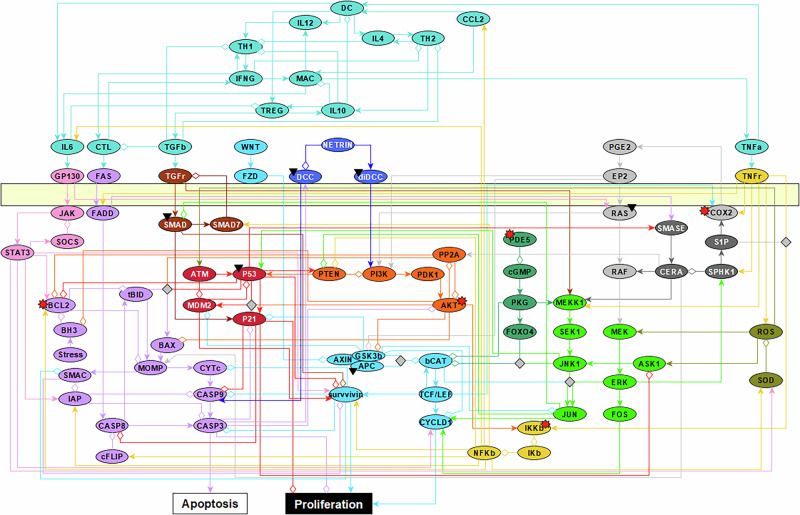
The nodes and their interactions in the spoCRC network. The network contains signaling cascades within the epithelial cell (all nodes below the yellow bar, which represents the plasma membrane) and signaling occurring in the cell’s external immune environment (nodes in turquoise). Activating relationships between nodes are illustrated by arrows. White diamonds indicate inhibitory relationships between nodes. Black triangles indicate nodes that are affected by mutations commonly occurring in CRC. The nodes with a red star are targets of COXIBs. Different colors were used to represent nodes with different biological functions. The nodes in turquoise belong to the immune-environment; the nodes in pink represent JAK/STAT signaling; the nodes in purple caspase signaling; the nodes in red p53 signaling; the nodes in brown SMAD signaling; the nodes in dark-blue netrin signaling; the nodes in blue WNT/β-catenin signaling; the nodes in orange AKT signaling; the nodes in yellow NF-κB signaling; the nodes in green MAPK signaling; the nodes in gray PGE2/COX2 signaling.

The spoCRC model developed in this study is based on a previously published Boolean model describing proliferation and survival of premalignant intestinal epithelial cells (IECS)^35^. To this model, we added several reactions enabling us to mimic the effect of genetic mutations detected in CRC or to simulate the on- and off-target effects of COXIBs (see Methods). The complete network includes 87 nodes and can be divided into two parts (Fig. 1): an intracellular part and an extracellular part. In the intracellular part, several signaling cascades are represented, including JAK-STAT signaling (Fig. 1 pink), Caspase signaling (Fig. 1, purple), WNT/β-catenin signaling (Fig. 1, blue), AKT signaling (Fig. 1, orange), NF-κB (Fig. 1, yellow), MAPK (Fig. 1, green), and COX/PGE2 signaling (Fig. 1, gray). These signaling cascades are connected to nodes representing apoptosis and proliferation of the epithelial cell (Fig. 1). The extracellular part of the network includes elements of the inflammatory microenvironment, such as immune cells (regulatory T cells: TREG; dendritic cells: DCs; Macrophages: MAC; Helper T-cells: TH), cytokines, and chemokines.

The spoCRC is constructed as a Boolean network, in which the network nodes can be either ON or OFF. The ON-state of a node represents the synthesis or activation of a protein/molecule, whereas the OFF-state represents either the inhibition or absence of a protein/molecule. Logical rules (and, or, not) define the relationships between nodes (see Supplementary Material 1). During simulation, nodes are selected randomly and it is evaluated whether the logical rules describing activation of the selected node are met. If the conditions are met, the node will be set to its ON-state. If the conditions are not met, the selected node will be set to its OFF-state. Because of randomness in node selection, simulations were repeated multiple times. From the simulation results, we calculated the activation frequency, which is defined as the number of simulations in which the node is ON divided by the total number of simulations performed. The higher the activation frequency of a node (maximum = 1), the more likely it is that the corresponding protein or cell is present in its activated form. In the case of the proliferation or apoptosis node, a high activation frequency implies that the phenotype of the epithelial cell is proliferative or apoptotic.

### The presence of the inflammatory network drives two positive feedback loops within the spoCRC network model

**Fig. 2:**
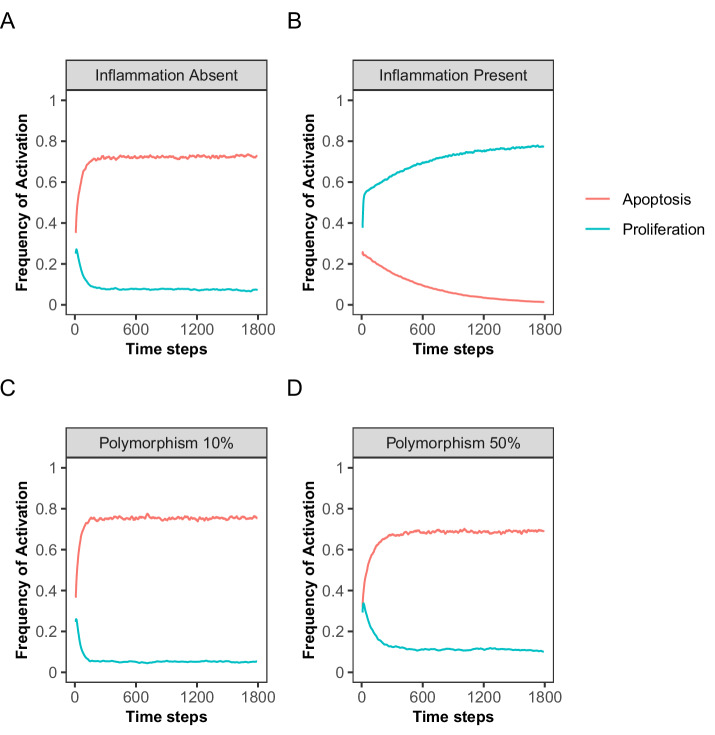
Effect of varying the strength of the inflammatory cluster on the frequency of activation of the proliferation and apoptosis nodes. **A** Simulation results for a model without the inflammation cluster (Inflammation absent, inflammatory nodes fixed to OFF-state), (**B**) with the inflammation cluster and complete activation of IL6 and CCL2 (Inflammation present, polymorphism set to 100%). **C**, **D** Simulation results in the presence of the inflammation cluster with the polymorphism values of IL6 and CCL2 set to 10% (**C**) and 50% (**D**). Plots show average over 5000 repetitions, with a running average of 20 time steps. Supplementary Fig. 2 shows the results for other polymorphism values tested.

Prior to investigating the effect of mutations frequently occurring in CRC, we investigated whether the model could recapitulate the relatively low proliferation rates of healthy IECS^36^. The Boolean model by Lu et al. (2015) showed that proliferation is highly dependent on the presence of inflammatory nodes. Thus, to obtain estimates of the minimal frequency of activation of the proliferation node, we executed the model in the absence of inflammatory nodes by fixing the corresponding nodes to the OFF state (See Methods). In the absence of the inflammation cluster, caspase signaling is highly active (average activation frequency over 5000 simulations; Caspase 3: 0.73, Caspase 9: 0.73, Supplementary Fig. 1). This leads to frequent activation of the apoptosis node (activation frequency: 0.73, Fig. 2A). The proliferation node is infrequently activated (activation frequency: 0.05, Fig. 2), which can be explained by a high frequency of p53 activation (activation frequency: 0.93). The activation frequency of the proliferation node is not equal to zero, because we allowed a basal activation level of the input nodes CAD, WNT, Netrin, and PDE5 by setting the polymorphisms of these nodes to 0.1, 0.1, 0.5, and 0.9, respectively (see Methods). This ensures low, but not zero, activity of β-catenin (average activation frequency: 0.04) and AKT (average activation frequency: 0.06) in the absence of inflammatory signaling. Thus, the Boolean model predicts low proliferation rates and relatively high rates of apoptosis in the absence of the inflammatory signaling.

We repeated the same analysis for a model in which the inflammation cluster was present (i.e. nodes not fixed to the OFF state but able to turn ON based on logic rules). Under those conditions, the activation frequency of the apoptosis node drops to almost zero (activation frequency: 0.01, Fig. 2B), while the proliferation node is frequently activated (activation frequency 0.80, Fig. 2B). The extreme effect of including the inflammatory network can be explained by two positive feedback loops involving the inflammatory nodes CCL2 and IL6. The cytokine IL6, which can be secreted by macrophages, dendritic cells (DCs) or the epithelial cell itself, activates the RAS pathway via the IL6 receptor GP130. In turn, RAS activates AKT, and subsequently NF-κB signaling. This leads to increased IL6 and CCL2 synthesis (Fig. 1). Through the activation of the AKT pathway, a second feedback loop gets activated. Within this positive feedback loop, AKT activation leads to the loss of inhibition of β-catenin. Via the COX2-PGE2 pathway, β-catenin increases the activity of RAS and AKT (Fig. 1). Thus, the AKT-pathway amplifies signaling through both positive feedback loops, leading to hyper-activation of the signaling pathways within the loops. The highly activated IL6 node (activation frequency >0.95) triggers the JAK/STAT pathway, which plays a dual role in cell regulation. It inhibits apoptosis via caspase inhibitors such as BCL2 and IAP, and promotes cell proliferation through the activation of cyclin D1 and survivin (Fig. 1). The hyper-activation of AKT, β-catenin and NF-κB (average activation frequencies: 0.98, 0.49, 0.98, respectively, supplementary Fig. 1) leads to a further promotion of proliferation and inhibition of apoptosis of the epithelial cell. Thus, via two positive feedback loops involving IL6, the presence of the inflammatory network leads to extreme activation of the proliferation node and inhibition of the apoptosis node. This extremely proliferative phenotype is not representative for the low basal proliferation rate of healthy microvili crypts^36^.

To prevent extreme activation of the positive feedback loops, we adjusted the polymorphism values of the nodes CCL2 and IL6. The polymorphism of a node determines the probability of activation of the node when the conditions of activation are satisfied and represents the stochasticity in the activation of a biological component. For instance, decreasing the polymorphism value of the IL6 node below 100% indicates that even if NF-κB is activated, transcription and translation of the IL6 gene to IL6 protein are not always occurring at a rate high enough to get sufficiently high extracellular IL6 concentrations. Polymorphism values < 100% will lead to a decrease in the activation frequency of the selected node. The polymorphism values tested for CCL2 and IL6 are 85%, 75%, 65%, 50%, 30% and 10%. At polymorphism values of 50% or less of the CCL2 and IL6 nodes, apoptosis is preserved and proliferation is kept low, consistent with non-tumorigenic behavior (Fig. 2C, D, Supplementary Fig. 2).

Taken together, this analysis showed that the presence of inflammatory signaling components within the spoCRC network allows activation of two positive feedback loops. This leads to strong inhibition of apoptotic signaling, whereas proliferation is promoted.

### Chronic inflammation inhibits apoptosis

**Fig. 3:**
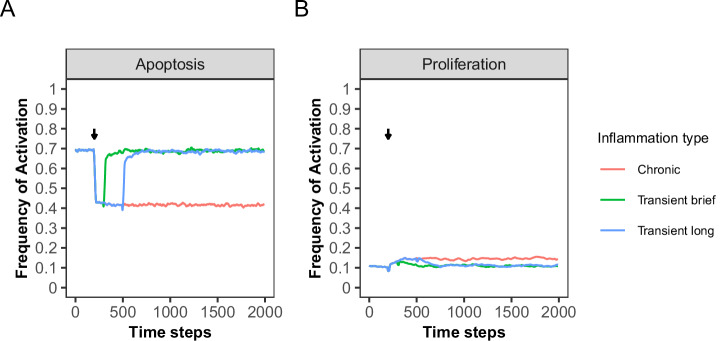
Impact of simulating chronic and transient inflammation on the activation frequency of the apoptosis and proliferation nodes. Activation frequency of the apoptosis (**A**) and proliferation (**B**) nodes in simulations representing chronic and transient inflammation. At time step 200 (indicated by the arrow), the DC node is fixed in its ON-state for either 100 (transient brief), 300 (transient long) steps or for the remainder of the simulation time (chronic). The first 200 time steps within this plot show the last time steps from healthy model simulations with the polymorphism values of IL6 and CCL2 set to 50% (see Fig. 2D). Average frequency of activation of the proliferation and apoptosis nodes is calculated over 5000 repetitions. Plots show running averages over 20 steps.

The above analysis showed that lower polymorphism values for IL6 and CCL2 in the model are necessary to reproduce the low proliferation rate of healthy IECS^36^. However, by lowering the polymorphism on IL6 and CCL2, the model was at risk of becoming unresponsive to inflammatory signals. Therefore, we analyzed the effect of inducing inflammation in those models by activating the dendritic cells (DCs), which have been found to create a pro-proliferative environment^35^. First, we explored a condition in which DCs were fixed in their ON state for the remainder of the simulation. We refer to this condition as chronic inflammation because of its continuous induction of inflammation. In the model with the polymorphism value set to 50%, fixing the DC node in its ON-state causes the activation frequencies of apoptosis to drop from 0.65 to 0.4 (Fig. 3A, Chronic inflammation). Simultaneously, a slight increase in the activation frequency of the proliferation was observed (Fig. 3B, Chronic inflammation). In models with polymorphism values of CCL2 and IL6 below 50%, continuous DC activation does not lead to changes in the activation frequency of the proliferation and apoptosis nodes (Supplementary Fig. 3). This indicates that at polymorphism values below 50% an inflammatory response cannot be triggered, not reflecting physiologically realistic conditions. Based on these findings, a polymorphism rate of 50% for both CCL2 and IL6 is selected for further model analysis.

To test the ability of the network to recover after inflammation, we fixed the DC node in the ON state for a duration of either 100 (brief transient inflammation) or 300 (long transient inflammation) steps, after which the DC node could become inactive. The frequency of activation of the apoptosis node recovers to baseline levels after both brief and long transient inflammation (Fig. 3A). In other words, using a polymorphism of 50% for both IL6 and CCL2, the spoCRC model can respond and recover from inflammation. Taken together, by setting the polymorphism value of IL6 and CCL2 to 50% our model is able to capture the behavior of healthy colon cells. We refer to those simulations as healthy model simulations.

### Mutations in signaling proteins promote cell survival and proliferation

**Fig. 4:**
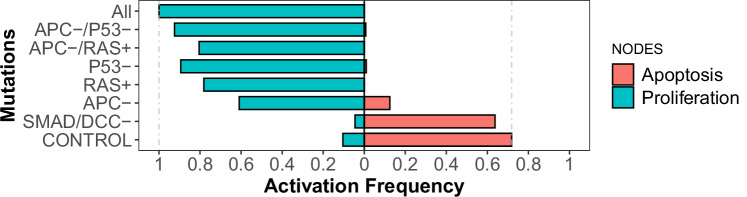
Effect of simulating various genetic mutations frequently occurring in CRC on proliferation and apoptosis of the epithelial cell. Activation frequency of the proliferation and apoptosis nodes is calculated from the activation status at the end of 5000 model simulations.

Having established a model representing signaling in cells without carcinogenic mutations, the spoCRC network was used to explore the mechanisms of CRC carcinogenesis. An initiating event in most sporadic cases of CRC is the occurrence of genetic mutations causing LOF of APC^34,37^. This occurs in at least 70% of the CRCs^33^. LOF mutations in the APC gene lead to a decreased binding affinity of the APC protein for the β-catenin deconstruction complex, consisting of APC, axin and glycogen synthase kinase 3 beta (GSK3B). Reduced binding to the deconstruction complex allows β-catenin to translocate to the nucleus. In our model simulations, APC LOF mutations are simulated by fixing the APC node in its OFF state. This causes increased activation of the β-catenin node (Table 1). Via activation of the CyclD1 and survivin nodes, β-catenin activates the proliferation node and inhibits apoptosis (Fig. 4). This effect is further amplified through activation of the COX2 node and subsequent activation of both positive feedback loops, which is evident from the increased activation frequency of the AKT and NF-κB nodes (Table 1).

**Table 1:** Frequency of activation of key nodes in the model with different mutations. *allelic loss of 18q is simulated by complete inhibition of SMAD, DCC and diDCC

Mutations				Activation frequencies nodes							Phenotype
18q*	APC	RAS	P53	Apoptosis	Proliferation	B-catenin	COX2/PGE2	AKT	NFKB	IL6	
				0.72	0.10	0.08	0.10	0.13	0.13	0.07	Apoptotic, Healthy simulations
X				0.64	0.04	0.05	0.06	0.05	0.05	0.03	Apoptotic
	X			0.12	0.61	0.50	0.57	0.74	0.76	0.38	Proliferative
		X		0.00	0.78	0.48	0.61	0.97	0.97	0.49	Proliferative
			X	0.01	0.89	0.40	0.51	0.85	0.87	0.45	Proliferative
	X	X		0.00	0.80	0.48	0.62	1.00	1.00	0.50	Proliferative
	X		X	0.01	0.92	0.48	0.59	0.88	0.89	0.44	Proliferative
X	X	X	X	0.00	1.00	0.49	0.62	1.00	1.00	0.50	Proliferative

In later stages of CRC carcinogenesis, activating mutations in KRAS and LOF mutations in p53 are frequently detected^33,34,37^. In our model simulations, continuous activation of RAS or inactivation of p53 has a similar effect as simulation of APC LOF (Fig. 4). Like APC LOF, overactivation of RAS directly drives both positive feedback loops. Also, fixing the p53 node in its OFF state leads to activation of the two positive feedback loops, as is evident from the strongly elevated activation frequency of the β-catenin, COX/PGE2, AKT, and NF-κB nodes (Table 1). This is because p53 inactivation leads to a direct loss of inhibition of the AKT pathway (Fig. 1).

Lastly, we explored the effect of allelic loss at chromosome 18q, which has been identified with high frequency in advanced stages of CRC^34,37^. Allelic loss at chromosome 18q was simulated by inactivation of the SMAD, DCC, and diCC nodes, as SMAD and DCC are located at 18q. In contrast to the above described simulations, inactivation of the SMAD and DCC (including diDCC) nodes has limited effect on apoptosis and proliferation (Fig. 3). Correspondingly, the inactivation of SMAD/DCC does not cause elevated activation of the nodes within the two positive feedback loops (Table 1, Supplementary Fig. 4). The lack of effect of SMAD/DCC inactivation contrasts clinical evidence associating SMAD4 loss with poor prognosis in CRC^38,39^, and is discussed in more detail in the discussion. Thus, our model simulations indicate that mutations in proteins that affect either of the two positive feedback loops have greater impact on the proliferation and survival of epithelial cells.

To simulate intermediate and late phases of CRC carcinogenesis, the combined effect of the above explored genetic mutations was simulated. Little additive effect is observed upon the combined loss of APC and p53 function, or the combination of APC LOF and RAS overactivation (Fig. 4), which represent intermediate stages of CRC carcinogenesis^34,37^. Late stages of CRC carcinogenesis were simulated by the combined inactivation of SMAD/DDC, p53, and APC and overactivation of RAS. These simulations show maximum activation of the proliferation node in the model (Fig. 4).

### Effects of inhibiting proteins targeted by COXIBs on proliferation and apoptosis in models containing CRC-associated mutations

**Fig. 5:**
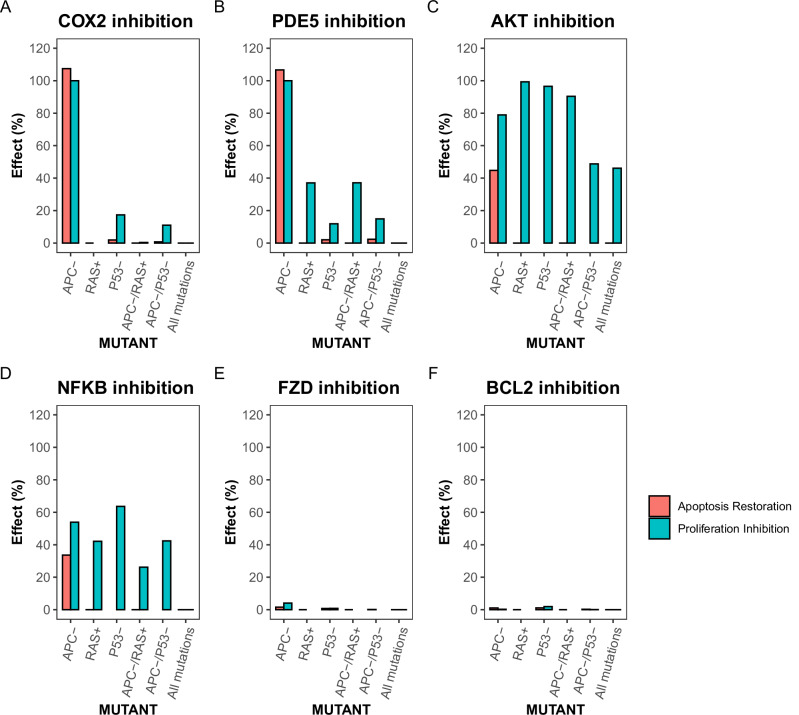
Effect of complete target inhibition on the restoration of apoptosis and inhibition of proliferation in simulations mimicking the effect of CRC-associated genetic mutations. The effect of COX2 (**A**), PDE5 (**B**), AKT (**C**), NF-κB (**D**), FZD (**E**) and BCL2 (**F**) inhibition on apoptosis and proliferation. For the calculation of ‘effect’ first the difference between frequency of node activation in the ‘treated’ vs ‘untreated’ simulation mimicking the presence of genetic mutations is calculated. This is then normalized to either the activation frequency of the apoptosis node in simulations without any mutations included or the activation frequency of the proliferation node in simulations mimicking the presence of genetic mutations. The condition ‘All mutations’ means the simulation is mimicking APC LOF, RAS GOF, P53 LOF, and SMAD and DCC LOF.

Next, we investigated how inhibition of the various COXIB targets influences apoptotic and proliferative signaling in simulations mimicking the presence of genetic mutations. In APC LOF simulations, complete inhibition of COX2 itself causes a restoration of apoptotic activity and inhibition of the proliferative phenotype (Fig. 5A). In this simulation, COX2 inhibition prevents the activation of AKT and NF-κB (Supplementary Table 1), leading to an inhibition of both positive feedback loops. Because COX2 is completely inhibited, whereas in ‘healthy’ simulations its activation frequency is >0, the activation frequency of the apoptosis node can increase above that of ‘healthy’ simulations, explaining the >100% restoration of apoptosis (Fig. 5A). In simulations mimicking the presence of any of the other genetic mutations, inhibition of COX2 is ineffective in restoring proliferative and apoptotic signaling (Fig. 5A). This unexpected finding can be explained by the ability of the mutated proteins to trigger activation of the AKT/NF-κB/IL6 positive feedback loop independently of COX2 activation (See Figs. 1, 7).

Inhibition of PDE5, a possible target of both celecoxib and sulindac^24,25^, has similar effects on the activation frequencies of the apoptosis and proliferation nodes (Fig. 5B). By increasing the cGMP levels, inhibition of PDE5 leads to an inhibition of β-catenin (Supplementary Table 1). Because β-catenin is the main driver of aberrant signaling in APC LOF simulations, the activation frequency COX2/PGE2, AKT and NF-κB are reduced to almost zero (Supplementary Table 1). For the other genetic mutations evaluated, overactivation of β-catenin is not the main cause of the elevated proliferation and survival, explaining why PDE5 inhibition has relatively little effect in those conditions (Fig. 5B).

Another target of celecoxib is AKT^23,29^. As with COX2 and PDE5 inhibition, inhibition of AKT in APC LOF simulations restores the activation frequencies of the apoptosis and proliferation nodes towards healthy levels, albeit the restoration is incomplete (Fig. 5C). For the other genetic mutations evaluated, inhibition of AKT causes a prominent decrease in the activation frequency of the proliferation node. Nonetheless, apoptotic signaling does not recover upon AKT inhibition (Fig. 5C).

NF-κB inhibition has similar effects as AKT inhibition in all conditions evaluated. However, in general, the effects of NF-κB inhibition are smaller (Fig. 5D). This is because NF-κB inhibition can only prevent activation of one of the two positive feedback loops. The activation frequency of the nodes in the other feedback loop, such as β-catenin, COX2 and AKT, remain elevated (Supplementary Table 1).

The last targets we evaluated are the WNT receptor FZD and the caspase inhibitor BCL2, which are suggested targets of both celecoxib and sulindac^21,26,32^. Both FZD and BCL2 do not directly affect the activity of either of the positive feedback loops (Fig. 1). It is therefore not surprising that FZD and BCL2 are ineffective in restoring apoptosis or inhibiting proliferation in any of the simulations mimicking the presence of genetic mutations. (Fig. 5E, F).

Thus, complete inhibition of proliferation and increased activation of apoptosis can be achieved by inhibiting COX2 or PDE5 in simulations incorporating LOF mutations in APC. As APC LOF is an early event in CRC carcinogenesis^34^, COX2 and PDE5 might be interesting targets during the early phases of disease development. In later phases of CRC carcinogenesis, in which various other genes get mutated, inhibition of AKT might be a more promising target to reduce the proliferation of CRC cells. However, as with any of the other drug targets evaluated, AKT inhibition can not restore apoptotic signaling in simulations representing intermediate or late phases of CRC development.

### Effects of celecoxib and sulindac on restoration of apoptosis and inhibition of proliferation in models incorporating CRC-associated mutations

Lastly, we investigated the effect of two COXIBs, celecoxib and sulindac, on the restoration of proliferation and apoptosis in early, intermediate and late phases of CRC. For each of the targets of the two COXIBs an inhibitory node was added to the network with a NOT relationship between the inhibitor and its target. In other words, when the inhibitory node is active, it will lead to an inactivation of the target. The activation frequency of the inhibitory nodes was set by polymorphism values < 100%, with higher polymorphism values leading to stronger target inhibition. Both COXIBs were assumed to almost completely block activation of COX2, by introducing a COX2 inhibition node with a polymorphism of 0.9. For simulating celecoxib, the polymorphism of the AKT, NF-κB, and PDE5 nodes was set to 0.3. Application of sulindac, and the eventual effect of sulindac’s active metabolite sulindac sulfide, was simulated by setting the polymorphisms of NF-κB and PDE5 to 0.3 and that of AKT to 0. This means that sulindac cannot inhibit AKT in our model simulations, following recent publications^21^. Possible differences between celecoxib and sulindac in potency to inhibit a specific target are not considered, as there is too little information available.

**Fig. 6:**
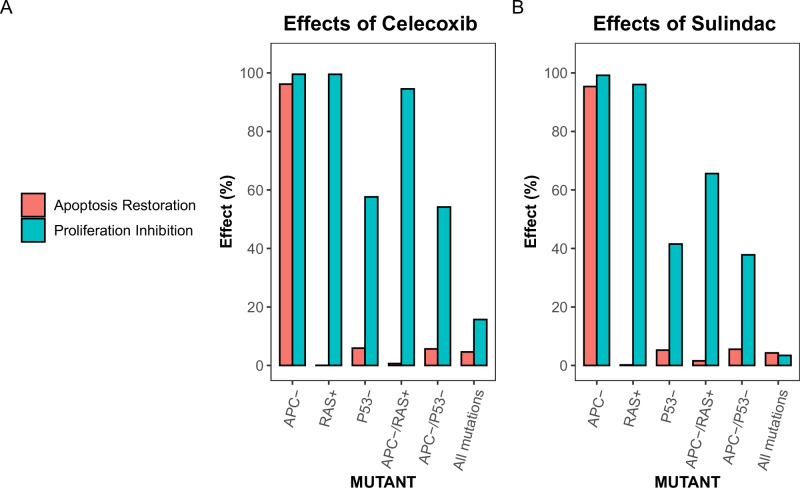
Effect of celecoxib and sulindac on the restoration of apoptosis and inhibition of proliferation in simulations mimicking the effect of CRC-associated genetic mutations. Restoration of apoptosis and inhibition of proliferation in simulations mimicking celecoxib (**A**) or sulindac (**B**) treatment. For the calculation of ‘effect’ first the difference between frequency of node activation in the ‘treated’ vs ‘untreated’ simulation mimicking the presence of genetic mutations is calculated. This is then normalized to either the activation frequency of the apoptosis node in simulations without any mutations included or the activation frequency of the proliferation node in simulations mimicking the presence of genetic mutations. The condition ‘All mutations’ means the simulation is mimicking APC LOF, RAS GOF, P53 LOF and SMAD and DCC LOF.

In APC LOF simulations, both celecoxib and sulindac restore the activation frequency of the apoptosis and proliferation nodes to rates observed in simulations performed in the absence of mutations (Fig. 6). In simulations representing intermediate stages of CRC development (simulations including LOF of P53, RAS overactivation, or the combination of APC LOF with either p53 LOF or RAS overactivation), celecoxib simulation shows a larger reduction in the frequency of activation of the proliferation node compared to sulindac (Fig. 6). As with the individual protein targets, neither celecoxib nor sulindac restores apoptosis in any condition other than loss of APC (Fig. 6). In conditions in which all tested proteins are mutated, representing the late stage of CRC, both celecoxib and sulindac have limited to no effect on apoptosis and proliferation (Fig. 6).

Taken together, in simulations other than those representing the effect of APC LOF, celecoxib is predicted to be more efficient compared to sulindac in preventing the proliferation of tumor cells. This difference is caused by the inhibitory activity of celecoxib on AKT. Other differences between the actions of sulindac and celecoxib are not considered in our model simulations.

## Discussion

In this work, we developed the spoCRC model to investigate the effect of COXIBs and their targets on the proliferation and survival of epithelial cells containing genetic mutations associated with CRC. The model is based on a previously published model^35^ and describes several intracellular signaling cascades involved in regulating cell proliferation and apoptosis. Additionally, the model includes components of the immune micro-environment, representing several cell types (such as dendritic cells, regulatory T-cells, and macrophages), chemokines, and cytokines.

**Fig. 7:**
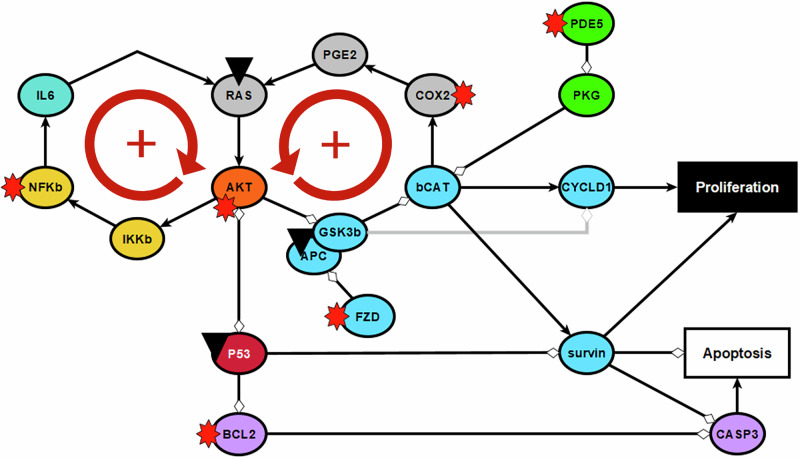
Schematic representation of the double positive feedback loop driving proliferation. Black triangles indicate nodes that are affected by CRC mutations. The nodes with a red star are targets of COXIBs.

Inflammation can play a major role in the development of CRC^3^. Correspondingly, in the spoCRC model, the immune micro-environment strongly promoted proliferation and survival (Fig. 2). In fact, we had to reduce the coupling between the immune micro-environment and the epithelial cell to prevent extreme proliferative and anti-apoptotic signaling. This was achieved by reducing the probability of activation of the IL6 and CCL2 nodes by 50% (i.e., setting their polymorphism values to 50%, Fig. 2). The impact of introducing polymorphisms on these nodes shows that those cytokines play an important role in coupling inflammation to aberrant proliferative signaling. In line with those findings, both cytokines are found to promote aberrant growth in tumor cells^40–43^, are highly expressed in CRC tumor cells^41,44–46^ and are associated with unfavorable prognosis of CRC patients^40,44^. In the spoCRC model, IL6 and CCL2 are part of a positive feedback loop, involving the activation of the RAS, AKT, and NF-κB pathways (Fig. 7). A second, and partially overlapping pathway, can be activated through the activation of AKT. In this pathway, AKT activation is followed by desinhibition of β-catenin, COX2 expression, PGE2 synthesis, activation of RAS, and subsequently AKT activation (Fig. 7). Activation of both positive feedback loops allows for increased proliferative signaling via β-catenin, survivin and p53 (Fig. 7). Additionally, by activating the JAK/STAT pathway, IL6 can inhibit apoptosis and promote proliferation (Fig. 1).

Several mutations found in CRC tumor cells, mutations in the APC, RAS, and p53 genes, directly or indirectly activate one of the proteins within the positive feedback loops (Fig. 7 black triangles). Thereby both feedback loops get highly activated leading to, for instance, elevated expression of IL6 and COX2, and aberrant activation of AKT (Table 1). Similar to the increased expression of IL6, elevated COX2 expression has been detected in samples of CRC patients and has been associated with reduced overall survival^19,20^. Furthermore, the aberrant activation of AKT is associated with tumorigenesis, cancer progression, and drug resistance in multiple human cancers, including CRC^27,28,47^. The activation of both positive feedback loops eventually leads to a strong promotion of proliferation and survival of the epithelial cell in simulations representing APC LOF, over-activation of RAS, and p53 LOF (Fig. 4). This result is in line with the strong proliferative phenotype observed in cell lines harboring one of these mutations^36,48,49^. In contrast to the mutations targeting a node within the two positive feedback loops, simulating the loss of chromosome 18q has limited impact on apoptosis and proliferation in the model (Fig. 4). This is inconsistent with multiple clinical and experimental studies showing that loss of SMAD4 correlates with increased tumorigenicity and poor overall survival of CRC patients^38,39^. The role of DCC, encoded also on 18q, in CRC is a matter of debate. Nonetheless, several studies indicate that DCC is an important tumor suppressor, especially when APC function is lost^50,51^. These observations suggest that our model may underestimate the contribution of SMAD4- and DCC-mediated signaling to the induction of apoptosis and suppression of proliferation in healthy contexts. Extending both down- and upstream (Netrin and TGF-β availability) signaling of the receptors might bring the model predictions closer to the observations.

When evaluating the effect of inhibiting proteins proposed to be targets of COXIBs, we found COX2 inhibition to be effective in reducing proliferation and promoting apoptosis of epithelial cells in simulations mimicking APC LOF. Simulations incorporating other CRC-associated mutations reveal NF-κB and, in particular, AKT as important COX-independent targets of celecoxib. AKT is a central node in both positive feedback loops. Corresponding to the central role AKT plays in aberrant proliferative signaling, various drugs targeting the PI3K/AKT pathway are evaluated in clinical trials for the treatment of CRC^27^. Nonetheless, AKT inhibition, as any of the other targets evaluated, can not restore apoptosis to normal rates in simulations incorporating the effect of genetic mutations other than mutations causing loss of APC function (Figs. 5, 6). This emphasizes the importance of early detection of adenomas for treatment efficacy, as APC LOF is considered as one of the earliest events in CRC development^34,37^. These findings align with the preventive effect of COXIBs^11,12,15^ and the limited clinical evidence supporting their efficacy at later disease stages^16^. Together, these results support the integration of genetic screening, in particular for APC LOF mutations, into surveillance programs of high-risk populations to guide targeted, preventive use of COXIBs before tumors progress to more advanced, treatment-resistant stages.

CRC, like all other cancers, is a highly complex disease involving many intracellular signaling cascades as well as interactions between the tumor and its environment. Although the spoCRC model could untangle part of it, the model does not capture the complete complexity of CRC carcinogenesis. Whereas the model focuses on the connection between signaling cascades and proliferation and apoptosis, cancer development also involves other processes, like migration and angiogenesis. Additionally, we evaluated only a specific set of gene mutations, all of which are included in the genetic model for colorectal tumorigenesis proposed by Fearon and Vogelstein^34^. Various other gene alterations have been detected in CRC samples^33,52^. Another limitation of the model is that the addition of inflammatory signaling components, which was crucial to uncover the two positive feedback loops, implied inclusion of processes occurring at different time-scales (e.g. binding of IL6 to its receptor occurs much faster compared to the recruitment of T-cells). These various time-scales were disregarded in the current implementation of the model. Future expansions of the model may be the inclusion of experimental information on the time-scales of the involved processes.

Taken together, the model provides several valuable insights guiding further experimental studies and drug development. We showed that COX-independent targets of COXIBs are crucial for their efficacy in the treatment of CRC. In particular, inhibition of the AKT pathway is an important effect of the COXIB celecoxib. Additionally, the model highlights the efficiency of drug targets to vary depending on the genetic mutations present in tumor cells. Such insights might be informative for patient stratification in future drug studies and suggest the benefit of genetic screening of early cancers.

## Methods

### The spoCRC network

The sporadic colorectal cancer (spoCRC) model has been developed based on the colitis-associated colon cancer (CAC) model developed by Lu et al.^35^ The well-documented Boolean CAC model contains many nodes relevant for sporadic CRC, including inflammatory signaling, COX/PGE, APC/β-catenin, RAS, AKT, NF-κB, and p53 signaling (Fig. 1). The signaling cascades within the CAC model regulate proliferation and apoptosis, which are used as the two main read-outs of the model simulations. To generate the spoCRC model, we added several reactions to the CAC model based on an extensive literature search.

One of the most important nodes added to the network is survivin. Survivin is a member of the inhibitor of apoptosis protein family, which, as its name implies, inhibits apoptosis by binding to caspases^53^. Additionally, survivin can suppress caspase activation by binding to SMAC^54^. In the spoCRC model, survivin was implemented to inhibit SMAC, CASP3, CASP9, and Cytochrome c (CYTc) (Supplementary Material 1). In addition to regulating apoptosis, survivin plays an important role in regulating multiple facets of cell division^53^, therefore we allowed survivin to activate the proliferation node (Fig. 1, Supplementary Material 1). The expression of survivin is mediated by many transcription factors, including NF-κB, STAT, and β-Catenin^55,56^, which are all nodes already included in the CAC model^35^. P53 and SMAD downregulate the transcription of survivin^53,57^, and were implemented to inhibit the survivin node in the spoCRC model (Supplementary Material 1). Lastly, we allowed survivin to get activated through MDM2^53^. Altogether, the addition of the survivin node allowed more signaling cascades to regulate proliferation and apoptosis compared to the original model.

We also added phosphodiesterase 5 (PDE5) signaling to the model (Fig. 1, Supplementary Material 1). PDE5 is one of the COX-independent targets of several COXIBs^21^. PDE5 degrades cGMP^58^, which at elevated levels activates protein kinase G (PKG) and subsequently Forkhead box protein O4 (FOXO4)^59^. Elevated FOXO4 signaling inhibits β-Catenin activity and thus proliferation^59^.

To investigate the effect of drugs targeting the WNT receptor, frizzled (FZD), WNT and FZD were added to the model (Fig. 1, Supplementary Material 1). Activated FZD inhibits Axin, which is part of the β-Catenin destruction complex (that also includes APC and GSK3-β)^60^.

Because of the clear link between disrupted β-catenin signaling and CRC^61^, we decided to extend the targets of this node in the model. β-catenin was implemented to activate the transcription factors TCF/LEF, and vice versa, representing the binding of both components to each other^62^. Additionally, we added activating interactions between TCF/LEF and its transcriptional targets already present in the original model: COX2^63^ survivin^56^, and Cyclin D1^64^.

To simulate loss of heterozygosity (LOH) of 18q, which contains the DCC gene, netrin signaling was added to the model. When not bound to netrin, the netrin receptor DCC activates caspase 9^65^. This was implemented in the model through an inhibitory interaction between the nodes netrin and DCC (Fig. 1, Supplementary Material 1). The netrin-bound DCC receptor, implemented in the model as diDCC (Fig. 1, Supplementary Material 1), can activate the PI3K/AKT pathway^65^.

Lastly, some minor changes to the CAC model were made. The BH3 node was added to the model. BH3 inhibits BCL2 and is activated by Stress^66^. Instead of direct activation of AKT by PI3K, PDK1 was implemented as a node that is activated by PI3K and subsequently activates AKT. Similarly, SEK1 was implemented to be activated by MEKK1 and to induce activation of JNK1, instead of a direct activation of JNK1 by MEKK1.

All reactions in the spoCRC model are illustrated in Fig. 1 and listed in Supplementary Material 1. An overview of the differences between the model developed by Lu et al.^35^ is given in Supplementary Table 2.

### Model implementation and simulation

The network is implemented in the form of a Boolean model, in which the network nodes can be either ON or OFF. Regulatory relationships between the nodes are represented by the logical operators: AND, OR, and NOT. The Boolean functions are described in Supplementary Material 1. To several nodes polymorphisms were added. The polymorphism of a node determines the probability of activation of the node when the conditions of activation are satisfied. Initially, polymorphisms were added for WNT, Netrin, CAD, PDE5, and stress (values: 0.1, 0.5, 0.1, 0.9, 0.02). These nodes represent inputs for various signaling cascades and ensure a basic level of proliferation. Later on also polymorphisms were added for IL6 and CCL2 (value: 0.5, see “Settings to simulate inflammation”).

At the start of the simulation, a random number determines whether a node is ON or OFF. An exception is that nodes in the inflammatory signaling cascade (including corresponding receptors), COX2, PGE2, EP2, as well as the apoptosis and proliferation nodes always start in the OFF state. During the model simulations, the nodes are updated following an asynchronous method, which is one of the general methods used in Boolean simulations. In this approach, in each iteration a random node is selected, which can switch the the ON-state when its logical conditions are met, or to the OFF-state when the logical conditions are not met. For instance, the AKT node, which activation is described by the logical rule: AKT = PDK1 &! (CASP3 | PP2A), will turn ON when PDK1 is ON and neither CAPS3 nor PP2A are ON. In any other condition to node will turn/stay OFF. This process continues for a given number of steps.

Because of the random nature of the method, multiple model simulations need to be performed to obtain an accurate estimation of the model behavior. In this work we used 5000 repetitions. For each node and each time-step, the average activation frequency was calculated over these repetitions. The average activation frequency is defined as the sum of all repetitions in which a node is in its ON state at a time-step divided by the total number of repetitions performed. Thus, an activation frequency of 1 implies that the node is always active, of 0 that it is inactive in all repetitions and of 0.5 that the node is active in half of the simulations.

Model simulations were performed in R (version 4.4.2.) using the library SPIDDOR (Version 1.0^67^,). Please note that in the asynchronous update method of SPIDDOR, multiple nodes can switch ON/OFF during a single time step.

### Settings to simulate inflammation

To simulate healthy cells in which APC is present, the APC node was fixed in its ON state. Nodes in the inflammation cluster were fixed in the OFF state, to simulate the model without the inflammatory network. For simulations in which the inflammatory network was present, various polymorphism values for IL6 and CCL2 were evaluated (100%, 85%, 75%, 65%, 50%, 30% and 10%). As polymorphisms of 50% for both IL6 and CCL2 resulted in normal proliferative and apoptotic signaling, and allowed the inflammatory network to become activated upon a trigger (DC over-activated, see below), for further simulations polymorphism of 50% was used.

To validate the network, we evaluated how the network responded to inflammation, which was simulated by over-activating dendritic cells (DC). This was done by fixing the DC node in its ON-state, following the chronic inflammation simulations performed using the CAC model^35^. For these inflammation simulations, the initial activation status for the various network nodes was determined randomly with a probability equal to the frequency of activation at the end of the healthy model simulation (number of steps = 1800, no DC over-activation). Transient inflammation was simulated by over-activation of the DC node for a short period of time-steps (100 or 300 steps for brief and long transient inflammation, respectively). The activation frequencies of the various nodes after brief or long transient inflammation were used as initial conditions for simulating the recovery phase. For these recovery simulations, the DC node was no longer fixed in its ON-state.

### Simulation of mutations

To simulate CRC, key mutations known to drive CRC progression were incorporated in the model. Loss-of-function mutations in the APC and p53 genes were simulated by fixing their respective nodes in the OFF state. Gain-of-function mutations in the RAS gene were simulated by fixing the RAS node in its ON state. 18q loss was simulated by fixing the nodes SMAD, DCC, and diDCC in their OFF state. For simulations in which individual gene mutations were investigated, the initial probability of node activation was determined by the frequency of activation of the nodes at the end of the ‘healthy’ model simulations. To simulate intermediate or late stages of CRC, in which multiple mutations were included in the model simulations, random initial conditions were based on the last steady state corresponding to APC mutant simulations only (to simulate combination of APC and p53 loss of function mutations, or RAS gain of function mutations), the combination of APC loss of function and RAS overactivation (to simulate the combination of APC loss of function, RAS overactivation and allelic loss at chromosome 18q), or the combination of APC loss, RAS overactivation and 18q loss (to simulate the combination of all mutations).

### Simulation of COXIBs

The effects of COXIBs on the frequency of activation of the proliferation and apoptosis nodes was evaluated by simulating total inhibition of COX2, PDE5, AKT, NF-κB, FZD and BCL2 (fixing the individual nodes in their OFF-state).

To simulate the effect of sulindac and celecoxib, which target the above-described nodes with different potency^21^, various additional nodes were added to the model (COXIB_AKT, COXIB_COX2, COXIB_NfkB, and COXIB_PDE5). These nodes were connected to their respective target via logical NOT statements, meaning that if the “COXIB_target” node were to get activated, the corresponding target would get inactivated. Different polymorphisms of the COXIB nodes were used to simulate the settings for sulindac and celecoxib. These polymorphisms are listed in Supplementary Table 3.

## Supplementary information

Supplementary information.

41540_2025_622_MOESM2_ESM.

## Data Availability

The R-scripts utilized to simulate and analyze the model are available in the supplementary material.

## Code availability

The R-scripts utilized to simulate and analyze the model are available in the supplementary material. In order to run the script the R-package SPIDDOR (version 1.0) is required^67^.
