# Supplementary material for: Network modeling of sporadic colorectal cancer reveals the importance of off-target effects of Cyclooxygenase inhibitors
Source: NPJ Syst Biol Appl. 2026 Feb 27;12:50. doi: 10.1038/s41540-025-00622-x (PMC13066020; doi:10.1038/s41540-025-00622-x)
Supplement: Supplementary file 1 — Supplementary information. [file 41540_2025_622_MOESM1_ESM.pdf]

Supplementary Material

Network modeling of sporadic colorectal cancer reveals the importance of off-target effects of Cyclooxygenase inhibitors

Supplementary Material 1 – Boolean functions of the spoCRC network

The spoCRC model contains the following boolean functions (Logical connections are indicated by & (AND), ! (NOT), and | (OR)):

AKT = PDK1 &! (CASP3 | PP2A)
Apoptosis = CASP3
ASK1 = ROS &! P21
ATM = ROS
AXIN = ! FZD
BAX = (PP2A & (P53 | TBID)) &! AKT
BCATENIN = (CAD | TCFLEF) &! ((FOXO4 & JNK) | PKG | (APC & AXIN & GSK3B))
BCL2 = (NFKB | STAT3) &! (BH3 | P53 | PP2A)
BH3 = STRESS
CASP3 = (CASP8 | CASP9) &! (IAP | SURVIVIN)
CASP8 = FADD &! (CFLIP | P21)
CASP9 = (CYTC | DCC) &! (IAP | P21 | SURVIVIN)
CCL2 = NFKB
CERAMIDE = SMASE &! SPHK1
CFLIP = NFKB
cGMP = ! PDE5
COX2 = TCFLEF | (S1P & TNFR)
CTL = IFNG &! TGFB
CYCLIND1 = (JUN | STAT3 | TCFLEF | FOS) &! GSK3B
CYTC = MOMP &! SURVIVIN
DC = (CCL2 | TNFA) &! IL10
DCC = CASP3 &! NETRIN
diDCC = NETRIN
EP2 = PGE2
ERK = MEK
FADD = FAS | TNFR
FAS = CTL
FOS = ERK
FOXO4 = PKG
FZD = WNT
GP130 = IL6
GSK3B = ! (AKT | EP2)
IAP = (NFKB | STAT3) &! SMAC
IFNG = CTL | TH1
IKB = ! IKK
IKK = AKT | (S1P & TNFR)
IL10 = TH2 | TREG
IL12 = DC | MAC
IL4 = DC | TH2
IL6 = DC | MAC | NFKB
JAK = GP130 &! SOCS

JNK = ASK1 | SEK1
JUN = (JNK & (BCATENIN | ERK)) &! GSK3B
MAC = (CCL2 | IFNG) &! IL10
MDM2 = (AKT & P53) &! (ATM | GSK3B)
MEK = RAF | ROS
MEKK1 = CERAMIDE | PKG | TGFR | TNFR
MOMP = (BAX | CERAMIDE | TBID) &! BCL2
NFKB = ! IKB
P21 = (P53 | SMAD) &! (CASP3 | GSK3B)
P53 = (ATM | JNK | PTEN) &! MDM2
PDK1 = PI3K
PGE2 = COX2
PI3K = (diDCC | EP2 | RAS) &! PTEN
PKG = cGMP
PP2A = CERAMIDE &! AKT
Proliferation = (SURVIVIN | CYCLIND1) &! (CASP3 | P21)
PTEN = P53 &! (JUN | NFKB)
RAF = CERAMIDE | RAS
RAS = EP2 | GP130
ROS = TNFR &! SOD
S1P = SPHK1
SEK1 = MEKK1
SMAC = MOMP &! SURVIVIN
SMAD = TGFR &! JUN
SMAD7 = NFKB | SMAD
SMASE = FADD | P53
SOCS = STAT3
SOD = NFKB | STAT3
SPHK1 = ERK | TNFR
STAT3 = JAK
SURVIVIN = (MDM2 | NFKB | STAT3 | TCFLEF) &! (P53 | SMAC | SMAD)
TBID = CASP8 &! BCL2
TCFLEF = BCATENIN
TGFB = TREG
TGFR = TGFB &! SMAD7
TH1 = (IFNG | IL12) &! (IL10 | IL4 | TGFB)
TH2 = IL4 &! (IFNG | TGFB)
TNFA = MAC
TNFR = TNFA
TREG = (DC | IL10) &! IL6
APC = 1
CAD = 1
NETRIN = 1
PDE5 = 1
STRESS = 1
WNT = 1

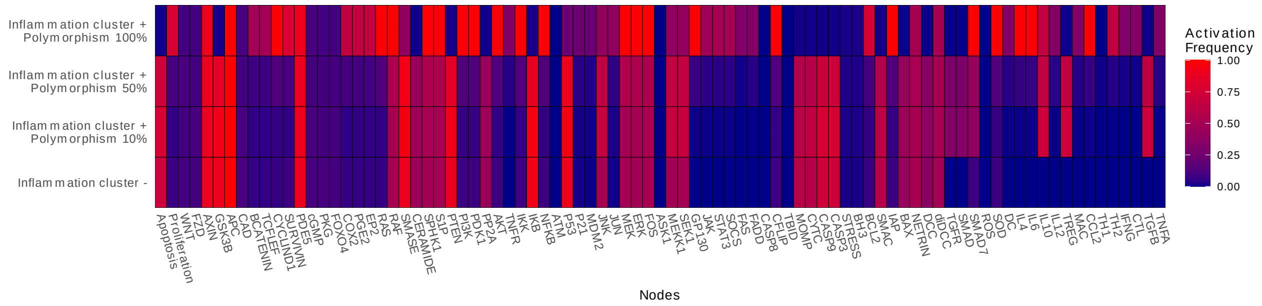

Figure S1: Effect of varying the strength of feedback through the inflammatory cluster on the frequency of activation of nodes in the spoCRC network. The heatmap shows the average frequency of activation of each node at the end of the simulation. Simulations are performed in the absence of the inflammation cluster (inflammation cluster -), and with the inflammation cluster (inflammation cluster +). The strength of the feedback occurring through the inflammation cluster was adjusted by setting the polymorphism values of the IL6 and CCl2 node to 10%, 50% and 100% polymorphism on IL6 and CCL2. Average activation frequency is calculated over 5000 repetitions.

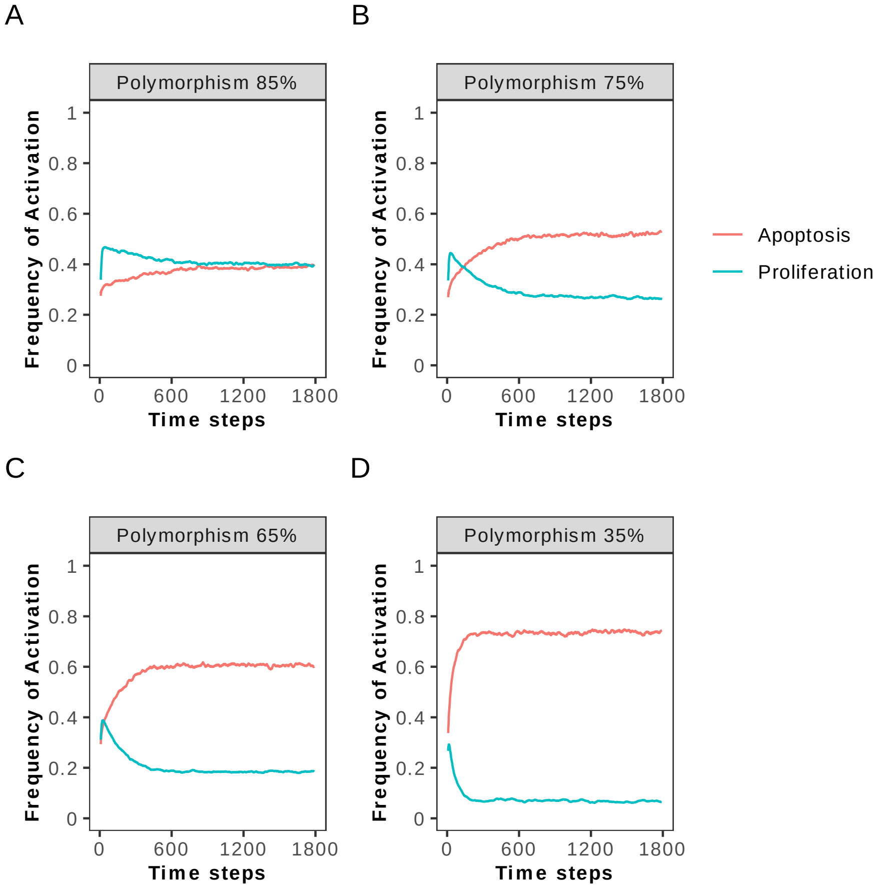

Figure S2: Frequency of activation of the apoptosis and proliferation nodes with polymorphism values of IL6 and CCL2 set to 85%, 75%, 35% or 65%. Plots show average over 5000 repetitions, with a running-average of 20 time steps.

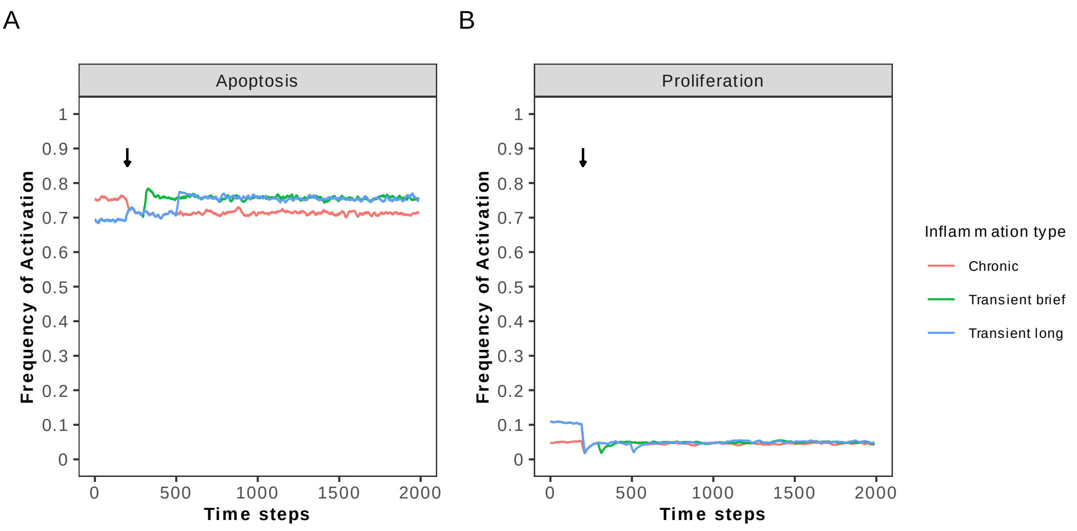

Figure S3: Impact of simulating chronic and transient inflammation on the activation frequency of the apoptosis and proliferation nodes when the IL6 and CCL2 have a polymorphism value of 10%. Activation frequency of the apoptosis (A) and proliferation (B) nodes in simulations mimicking chronic and transient inflammation. At time step 200 (indicated by the arrow), the DC node is fixed in it's ON-state for either 100 (transient brief), 300 (transient long) steps or for the remainder of the simulation time (chronic). The first 200 time steps within this plot show the last time steps from healthy model simulations with the polymorphism values of Il6 and CCL2 set to 10%. Average frequency of activation of the proliferation and apoptosis nodes is calculated over 5000 repetitions. Plots show running averages over 20 steps.

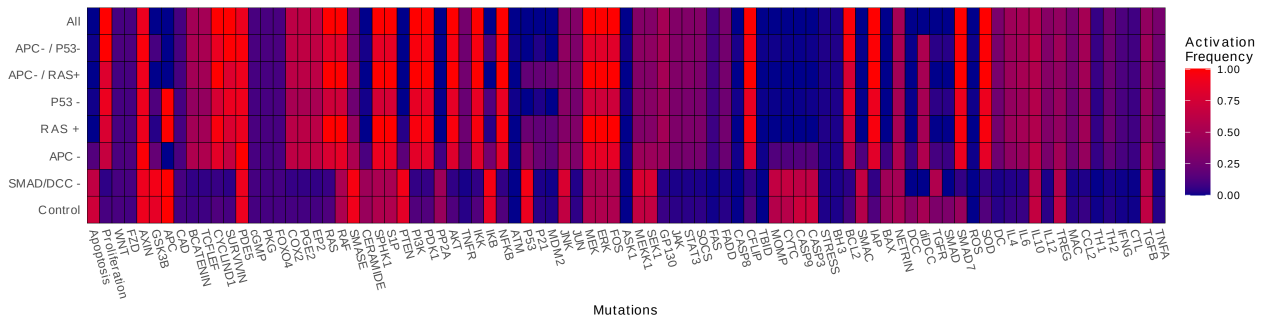

Figure S4: Frequency of node activation in simulations incorporating different mutations into the spoCRC model. The heatmap represent the average frequency of activation of the various nodes in the network at the end of the simulation.

Table S1: Frequency of activation of key nodes after inhibition of proposed COXIB-targets in simulations incorporating the effect various genetic mutations associated to CRC.

**Mutations**				**Inhibitor targets**						**Activation frequencies nodes**						
**APC**	**RAS**	**P53**	**18q**	**COX2**	**PDE5**	**AKT**	**NFKB**	**FZD**	**BCL2**	**Apoptosis**	**Proliferation**	**B-catenin**	**COX2/PGE2**	**AKT**	**NFKB**	**IL6**
X										0.12	0.61	0.5	0.57	0.74	0.76	0.38
X				X						0.83	0	0.49	0	0	0	0
X					X					0.82	0	0	0	0	0	0
X						X				0.42	0.13	0.48	0.49	0	0	0
X							X			0.35	0.28	0.5	0.5	0.47	0	0
X								X		0.14	0.58	0.48	0.57	0.72	0.74	0.38
X									X	0.13	0.61	0.49	0.57	0.73	0.74	0.38
	X									0	0.78	0.48	0.61	0.97	0.97	0.49
	X			X						0	0.8	0.49	0	1	1	0.49
	X				X					0	0.49	0	0.26	1	1	0.5
	X					X				0	0.01	0.03	0.04	0	0	0
	X						X			0	0.45	0.41	0.41	0.85	0	0
	X							X		0	0.82	0.5	0.63	1	1	0.5
	X								X	0	0.81	0.48	0.61	1	1	0.5
		X								0.01	0.89	0.4	0.51	0.85	0.87	0.45
		X		X						0.02	0.74	0.18	0	0.66	0.69	0.37
		X			X					0.02	0.79	0	0.16	0.73	0.76	0.39
		X				X				0	0.03	0.03	0.03	0	0	0
		X					X			0	0.33	0.29	0.29	0.65	0	0
		X						X		0.01	0.89	0.4	0.5	0.85	0.87	0.44
		X							X	0.02	0.88	0.41	0.51	0.85	0.86	0.44
X	X									0	0.8	0.48	0.62	1	1	0.5
X	X			X						0	0.8	0.51	0	1	1	0.5
X	X				X					0	0.51	0	0.26	1	1	0.5
X	X					X				0	0.08	0.48	0.48	0	0	0
X	X						X			0	0.59	0.47	0.48	0.97	0	0
X	X							X		0	0.81	0.49	0.62	1	1	0.51
X	X								X	0	0.81	0.5	0.63	1	1	0.5
X		X								0.01	0.92	0.48	0.59	0.88	0.89	0.44
X		X		X						0.01	0.82	0.5	0	0.67	0.71	0.37
X		X			X					0.02	0.79	0	0.15	0.72	0.75	0.4
X		X				X				0	0.47	0.47	0.48	0	0	0
X		X					X			0	0.53	0.49	0.5	0.76	0	0
X		X						X		0.01	0.93	0.49	0.6	0.89	0.89	0.44
X		X							X	0.01	0.92	0.5	0.59	0.87	0.89	0.46
X	X	X	X							0	1	0.49	0.62	1	1	0.5
X	X	X	X	X						0	1	0.49	0	1	1	0.49
X	X	X	X		X					0	1	0	0.26	1	1	0.49
X	X	X	X			X				0	0.54	0.48	0.48	0	0	0
X	X	X	X				X			0	1	0.47	0.48	1	0	0
X	X	X	X					X		0	1	0.5	0.62	1	1	0.5
X	X	X	X						X	0	1	0.51	0.64	1	1	0.5

Table S2: Logic rules differing between CAC model published by Lu et al., (2015) and the spoCRC model.

CAC model	spoCRC model
AKT = PI3K &! (CAPS3 | PP2A)	AKT = PDK1 &! (CASP3 | PP2A)<br>PDK1= PI3K<br>FZD =WNT<br>AXIN =! FZD
BCATENIN = !(APC&GSK3B)	BCATENIN =(CAD |TCFLEF) &! ((FOXO4 & JNK) | PKG | (APC & AXIN & GSK3B))<br>TCFLEF= CATENIN
COX2 = S1P & TNFR	COX2 = TCFLEF | (S1P & TNFR)
CYCLIND1 =(JUN | STAT3) &! GSK3B	CYCLIND1= (JUN | STAT3|TCFLEF|FOS)&! GSK3B<br>BH3=STRESS
BCL2 = (NFKB | STAT3) &! (P53 | PP2A)	BCL2=(NFKB | STAT3) &! (BH3 | P53 | PP2A)<br>SURVIVIN = (MDM2 | NFKB |STAT3 |TCFLEF) &! (P53 | SMAC | SMAD)
CASP3 = (CASP8 | CASP9) &! IAP	CASP3 = (CASP8 | CASP9) &! (IAP | SURVIVIN)
CASP9 = CYTC &! (IAP | P21)	CASP9 = (CYTC | DCC)&! (IAP | P21 | SURVIVIN)
Proliferation = CYCLIND1) &! (CASP3|P21)	Proliferation = (SURVIVIN | CYCLIND1) &! (CASP3 | P21)
SMAC = MOMP	SMAC = MOMP &! SURVIVIN
CYTC = MOMP	CYTC = MOMP &! SURVIVIN<br>cGMAP =! PDE5<br>PKG = cGAMP<br>FOXO4 = PKG<br>DCC = CASP3 &! NETRIN<br>diDCC = NETRIN
PI3K = (EP2 | RAS) &! PTEN	PI3K = (diDCC | EP2 | RAS) &! PTEN<br>SEK1= MEKK1
JNK = ASK1 | MEKK1	JNK = ASK1 | SEK1

Table S3: Polymorphism values of the COXIB_target nodes used to simulate sulindac and celecoxib.

Target	Celexocib	Sulindac
COX2	0.9	0.9
AKT	0.3	0
NFKB	0.3	0.3
PDE5	0.3	0.3

Lu, Junyan, Hanlin Zeng, Zhongjie Liang, et al. 2015. “Network Modelling Reveals the Mechanism Underlying Colitis-Associated Colon Cancer and Identifies Novel Combinatorial Anti-Cancer Targets.” *Scientific Reports* (England) 5 (October): 14739. https://doi.org/10.1038/srep14739.
